# Artificial intelligence-enabled electrocardiographic ‘sex discrepancy’ as a predictor of atrial fibrillation recurrence: contextualising the findings of park *et al.*

**DOI:** 10.1093/ehjdh/ztaf110

**Published:** 2025-09-22

**Authors:** Panteleimon Pantelidis, Emmanouil Charitakis, Evangelos Oikonomou

**Affiliations:** 3rd Department of Cardiology, Sotiria Hospital, National and Kapodistrian University of Athens, 152 Mesogeion Street, Athens 115 27, Greece; Department of Cardiology, Karolinska University Hospital, Stockholm, Sweden; 3rd Department of Cardiology, Sotiria Hospital, National and Kapodistrian University of Athens, 152 Mesogeion Street, Athens 115 27, Greece

Artificial Intelligence (AI)-enabled analysis of 12-lead electrocardiograms can recover latent phenotypes, such as age and sex, that show emerging prognostic value across cardiovascular conditions.^1–3^ Park *et al*.^4^ extend this notion by examining an AI-derived ‘sex discrepancy’, defined as cases in which AI-enabled ECG analysis assigns a probability >0.5 to the opposite biological sex, classifying the patient as ‘discrepant’, vs. ≤0.5, classified as ‘non-discrepant’. In their analysis, this AI-ECG sex discrepancy was associated with higher rates of atrial fibrillation (AF) recurrence following catheter ablation.^4^ This observation raises two tractable questions: (i) could subgroup performance and class balance influence the apparent effect size, motivating per-sex calibration and augmented fairness-aware reporting, and (ii) is the signal-derived sex category acting as an independent predictor, or rather as a surrogate for biological aging, for example, advanced atrial remodelling and other established determinants of AF outcomes with known disparities between men and women?^5^

Turning the ECG reflected trait ‘sex discrepancy’ into a lens on post-ablation risk seems a genuinely fresh idea. In the paper, Park and colleagues^4^ deliver an AI model that performs strongly in determining sex in external datasets (area under the curve, AUC 0.89–0.92). The AI-predicted sex disparities are associated with 5-year AF recurrence, yet, crucially, only in women (hazard ratio, HR 1.42) and not in men (HR 1.01).^4^ Rather than contest this finding, we see an opportunity to refine how the signal might be understood and how it might be used under this scope.

At first glance, this women-specific asymmetry seems to function as a surrogate for atrial health. The authors’ own data suggest that sex-discrepant women were older and exhibited larger left atrial diameters as well as higher E/e′ values. Even heart failure prevalence was numerically higher (15% vs. 11%, though not statistically significant). Most importantly, in women, sex discrepancy was significantly associated with left atrial enlargement (adjusted odds ratio, OR 1.67), with a median left atrial diameter of 41 mm (interquartile range, IQR 37–45) in discrepant vs. 38 mm (IQR 34–42) in non-discrepant cases. In men, no such association was observed (adjusted OR 0.88), with a median diameter of 39 mm (IQR 34–43) in discrepant vs. 40 mm (IQR 36–43) in non-discrepant cases. That pattern fits what we already know: women referred for ablation often carry greater diastolic burden and more advanced atrial remodelling, with higher recurrence risk,^5^ and left atrium size/volume and diastolic indices (E/e′) are reproducible predictors of late recurrence after AF ablation.^6,7^ Notably, as subgroup analysis dictates (see Supplementary material online, *Table S3*),^4^ the model tends to perform even better in younger, non-diabetic women with ‘healthier hearts’, as reflected by a lower atrial diameter and E/e′ index. This may indicate that the ‘sex discrepancy’ marker delineates a subgroup of women whose atrial and overall phenotypic profile more closely resembles that typically seen in men, and hence the model’s tendency to classify them toward the ‘male’ side. In doing so, it may be collectively and effectively capturing additional factors that drive AF recurrence beyond the conventional predictors absent in this subgroup. This explanation underscores the importance of the findings and helps place them into context. If AI-ECG sex discrepancy acts as a surrogate marker of atrial health and a deviating phenotype in women, it can hold clinical value, provided that we can determine its incremental contribution beyond traditional risk factors. Advanced modalities such as cardiac magnetic resonance imaging (MRI) and electroanatomical mapping could offer complementary insights to uncover the underlying substrate in this direction.

In addition, this well-defined association, reflected by the significant HR, warrants further evaluation before it can be translated into practical usefulness. In women, the C-index for AI-ECG sex probability is ∼0.535, similar to age and other features’ discriminative power, and lower than that of left atrium diameter alone (∼0.538).^4^ By convention, a C-index near 0.53 is only marginally above chance, indicating limited value for stand-alone risk stratification. This sets the basis and underscores the value for future work to clarify clinical utility. Calibration metrics, like calibration-in-the-large or calibration slope, as well as fairness-aware metrics in line with current guidance,^8^ can elaborate better on the ‘areas’ of patient distribution where the model seems to predict best, ensuring that observed effects are not artefacts. Second, the incremental value of this novel, promising ‘sex discrepancy’ signal over established predictors merits further exploration. Beyond the factors already included in the regression models (see Supplementary material online, *Table S3*^4^), such as age, left atrial diameter, and E/e′, future analyses could incorporate additional clinical and imaging parameters, including AF type, left atrial volume and strain, and more advanced MRI- and mapping-derived markers, which remain unexplored in this context. Collinearity metrics can confirm the absence of redundancy with conventional markers, and, at the same time, measures such as net reclassification improvement and decision-curve analysis can quantify whether the AI-ECG sex discrepancy meaningfully improves patient-level risk stratification and clinical decision-making. Building a composite ‘biologic atrial age’ marker, combining imaging-derived atrial metrics (volume, strain, fibrosis in MRI), AI-ECG age as previously proposed by the same research group,^9^ along with AI-ECG sex discrepancy probability given that its incremental value is established, could more comprehensively reflect the risk of recurrence after ablation.

In short, what Park and colleagues have discovered may function as a stethoscope for atrial health in women, heard through the ECG and translated by AI. The ‘female-only’ association is plausibly explained by differences in atrial- and patient-level phenotypes among women with recurrence, and it reveals the presence of unexplored factors that call for further investigation. Demonstrating subgroup-robust behaviour, quantifying additive value, and externally validating in broader settings, particularly in persistent AF and multi-ethnic cohorts under standardized lesion sets and monitoring protocols, will be essential to clarify transportability and clinical deployment. In this light, the authors’ work should be praised as an important step forward, and its continued exploration will be of considerable interest.
